# Supplementary material for: Screening non-conventional yeasts for acid tolerance and engineering Pichia occidentalis for production of muconic acid
Source: Nat Commun. 2023 Aug 31;14:5294. doi: 10.1038/s41467-023-41064-5 (PMC10471774; doi:10.1038/s41467-023-41064-5)
Supplement: Supplementary file 1 — Suplementary Information [file 41467_2023_41064_MOESM1_ESM.pdf]

Screening non-conventional yeasts for acid tolerance and engineering *Pichia occidentalis* for production of muconic acid

Pyne *et al*.

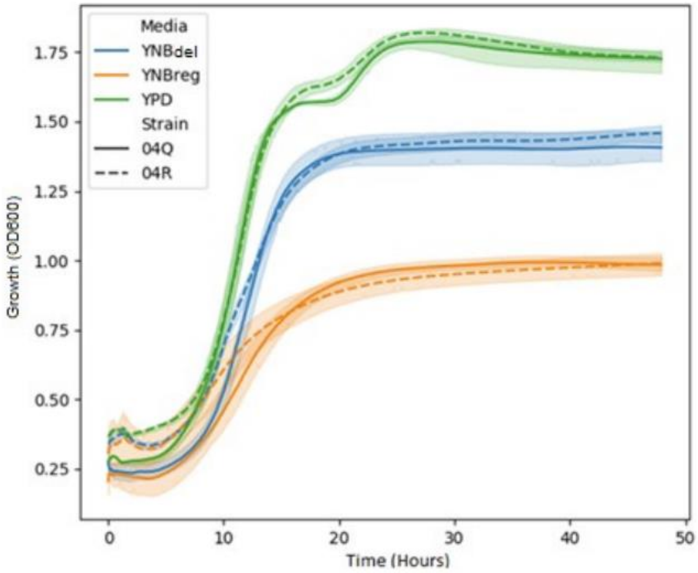

Supplementary Figure 1. Comparison of *P. occidentalis* strain 04Q (YB-3389) and 04R (Y-6545) growth in standard 1× YNB medium (YNBreg) vs growth in 3× YNB medium (YNBdel). Highlighted area shows the standard deviation of $n = 3$ independent biological samples. YNBreg contains 20 g L^{-1} glucose, 5.1 g L^{-1} ammonium sulfate and 1.7 g L^{-1} Yeast Nitrogen Base. YNBdel contains 20 g L^{-1} glucose, 5.1 g L^{-1} ammonium sulfate and 5.1 g L^{-1} Yeast Nitrogen Base.

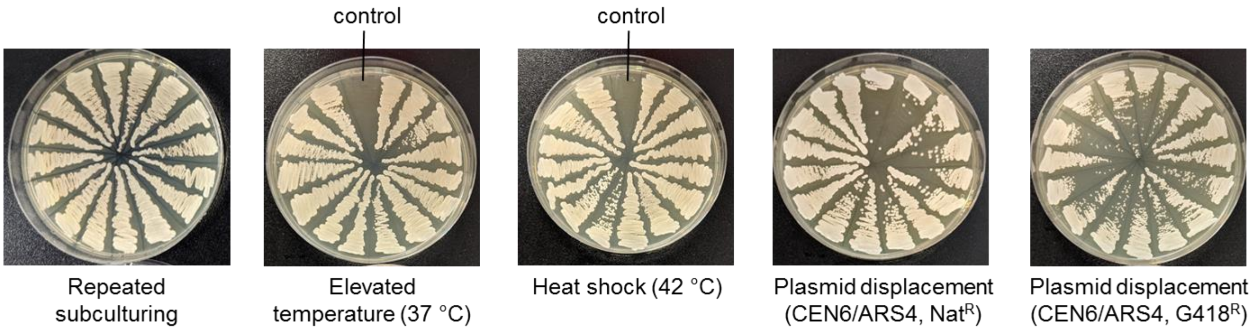

Supplementary Figure 2. Attempted curing of plasmid pCas-Hyg-CEN6ARS4-ADE2 from *P. occidentalis* Y-7552. Various curing methods were assessed to cure cells of the pCas-Hyg-ADE2 plasmid. Hyg^R colonies were subcultured six times without selection at 30 °C (repeated subculturing) or 37 °C (elevated temperature) prior to screening colonies on YPD agar plates containing hygromycin. Curing was also attempted by heat-shocking cells at 42 °C in a mock lithium-acetate-PEG transformation (heat shock) or by attempting to displace the original pCas-Hyg-ADE2 plasmid with Nat^R or G418^R derivatives containing the same CEN6/ARS4 origin. Wild-type *P. occidentalis* lacking a pCas plasmid was included as a control.

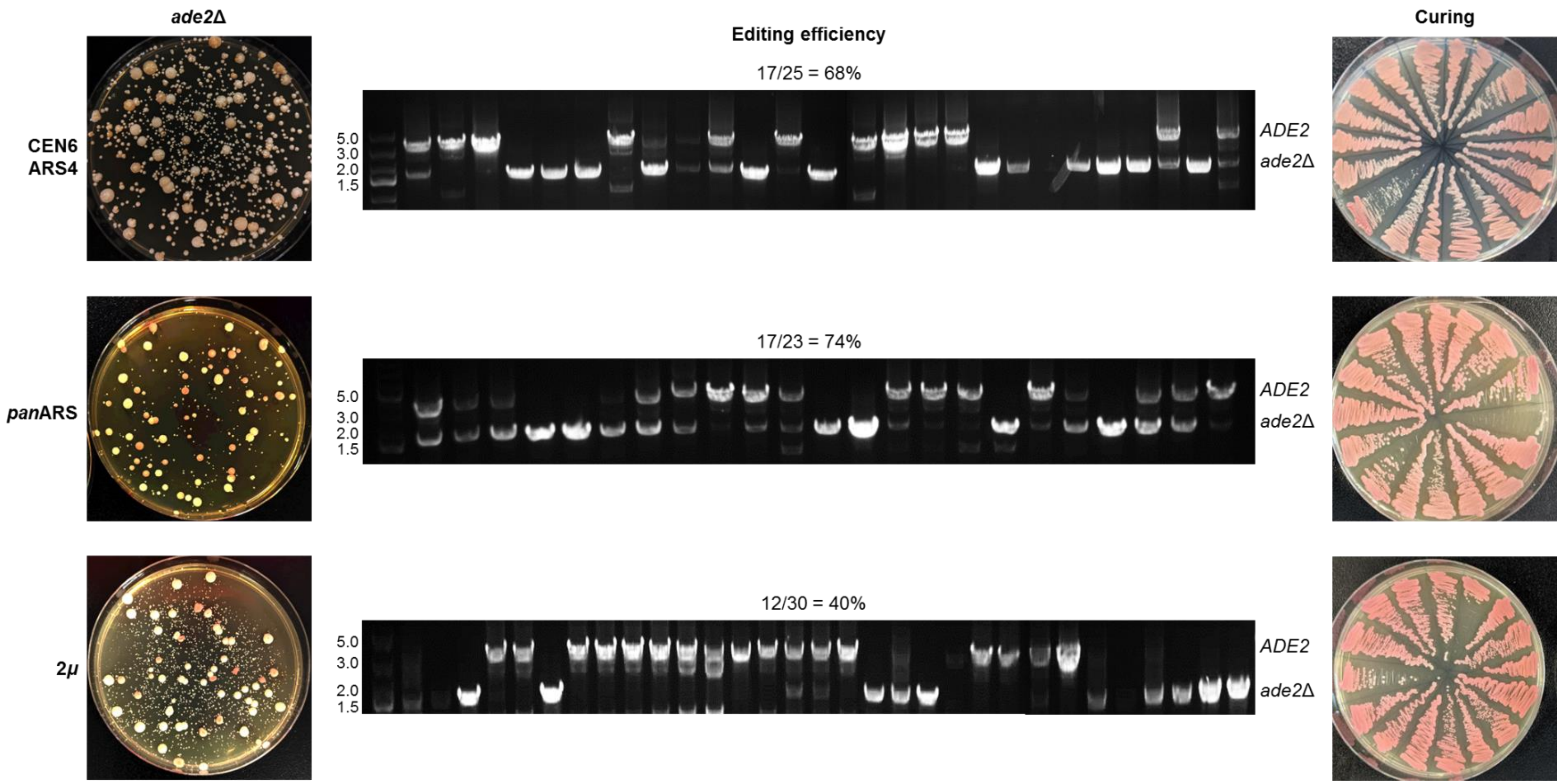

Supplementary Figure 3. Deletion of *ADE2* in *P. occidentalis* Y-7552 using various pCas plasmid origins. The *P. occidentalis ADE2* gene was deleted using a pCas-Hyg-ADE2 plasmid containing a CEN6/ARS4, *pan*ARS, or 2*μ* origin. Editing efficiency is shown based on screening of 23-30 random colonies. One *ade2*Δ mutant colony containing each plasmid origin was subcultured six times in YPD without selection and resultant colonies were screened for loss of Hyg^R on YPD agar plates containing hygromycin. Repeating *ADE2* deletion using different plasmid origins routinely yielded similar editing efficiencies. Source data are provided as a Source Data file.

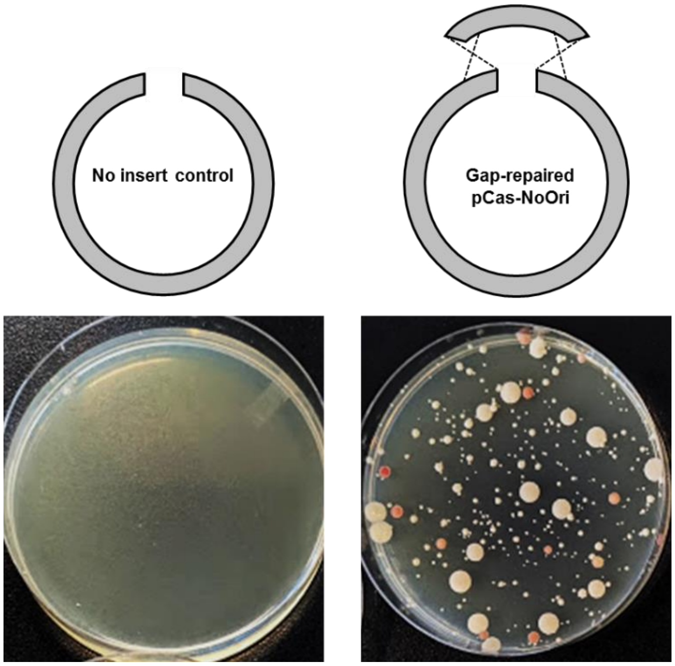

Supplementary Figure 4. Deletion of *ADE2* in *P. occidentalis* Y-7552 without a plasmid origin. The *P. occidentalis ADE2* gene was deleted using a pCas-Hyg-ADE2 plasmid lacking a plasmid origin. Plasmid pCas-Hyg-ADE2 was digested within the CEN6/ARS4 origin and the linearized plasmid was used to transform *P. occidentalis* along with an overlapping gap repair template lacking a plasmid origin. The resulting origin-less pCas plasmid generated red colonies following selection on YPD agar containing hygromycin. Omitting a linear gap repair template failed to generate Hyg^R transformants (no insert control).

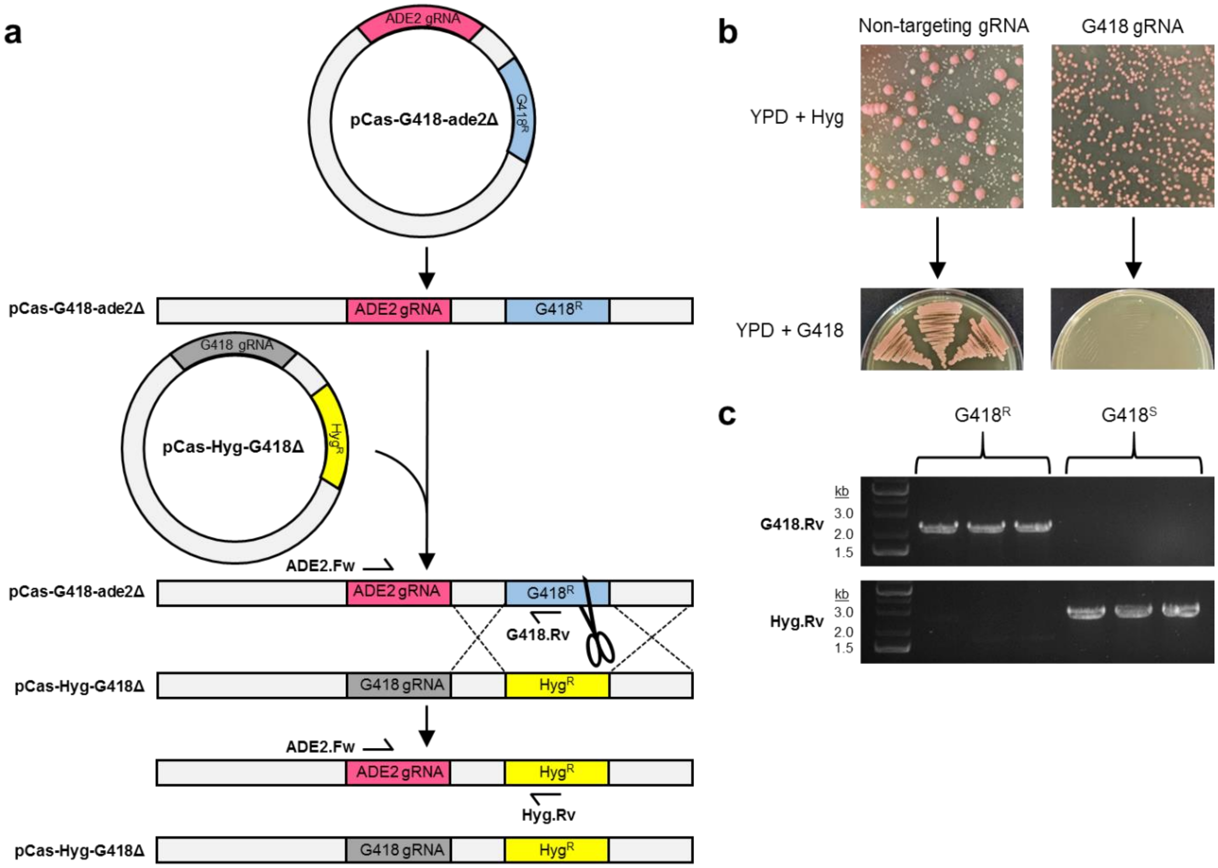

Supplementary Figure 5. Plasmid integration and antibiotic marker recycling in *P. occidentalis* Y-7552. a, Proposed mechanism of plasmid integration and antibiotic marker recycling. Introduction of plasmid pCas-G418-ade2Δ and selection of G418^R colonies yields pink *ade2*Δ colonies upon integration of pCas-G418-ade2Δ. Transformation of a pink G418^R *ade2*Δ mutant with plasmid pCas-Hyg-G418Δ harboring a Hyg^R marker and a gRNA targeting chromosomal G418^R yields Hyg^R colonies upon chromosomal integration of pCas-Hyg-G418Δ. Transcription of the G418 gRNA introduces a double stranded DNA break to the chromosomal G418^R marker, which is repaired by the homologous chromosomal Hyg^R marker. The resulting strain lacks a G418^R marker. Primers used for PCR screening in **c** are shown. **b,** Introduction of a gRNA targeting a chromosomal G418^R marker yields a substantial increase in Hyg^R transformants compared to a control transformation utilizing a non-targeting gRNA (top). In line with the mechanism outlined in **a**, Hyg^R colonies transformed with G418 gRNA lost G418^R, while colonies transformed with a non-targeting gRNA retained G418^R (bottom). **c,** Colony PCR confirmation of the proposed marker swapping mechanism outlined in **a**. Screening G418^S colonies using primers ADE2.Fw + G418.Rv confirms loss of the chromosomal G418-resistance marker. Screening G418^S colonies using primers ADE2.Fw + Hyg.Rv confirms acquisition of the Hyg^R marker not observed in G418^R colonies transformed with a non-targeting gRNA. Three representative G418^R and G418^S colonies were screened. Source data are provided as a Source Data file.

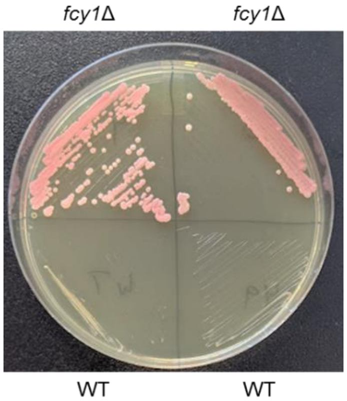

Supplementary Figure 6. Deletion of *FCY1* confers resistance to 5-fluorocytosine (5-FC). *FCY1* was deleted in an *ade2*Δ G418^{R} host using pCas-Hyg-CEN6ARS4-PoADE2. Transformants were restreaked onto YPD agar plates overlaid with 400 μl of a 10 g L^{-1} solution of 5-FC.

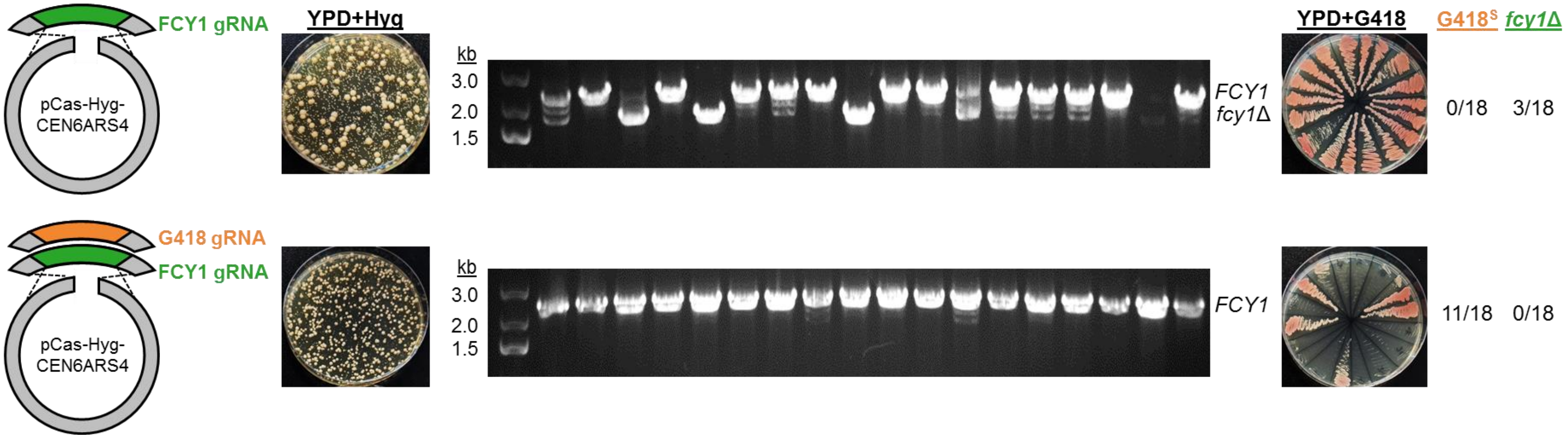

Supplementary Figure 7. Deletion of *FCY1* and G418^{R} marker recycling in *P. occidentalis* Y-7552 using dual gRNA species. Dual gRNA species targeting a chromosomal G418^{R} marker and the *FCY1* gene were introduced to a G418^{R} *ade2*Δ mutant (bottom) and compared to a control transformation using a single *FCY1* gRNA (top). Hyg^{R} transformants were scored for *fcy1*Δ by colony PCR and G418^{S} by restreaking transformants onto YPD+G418. Source data are provided as a Source Data file.

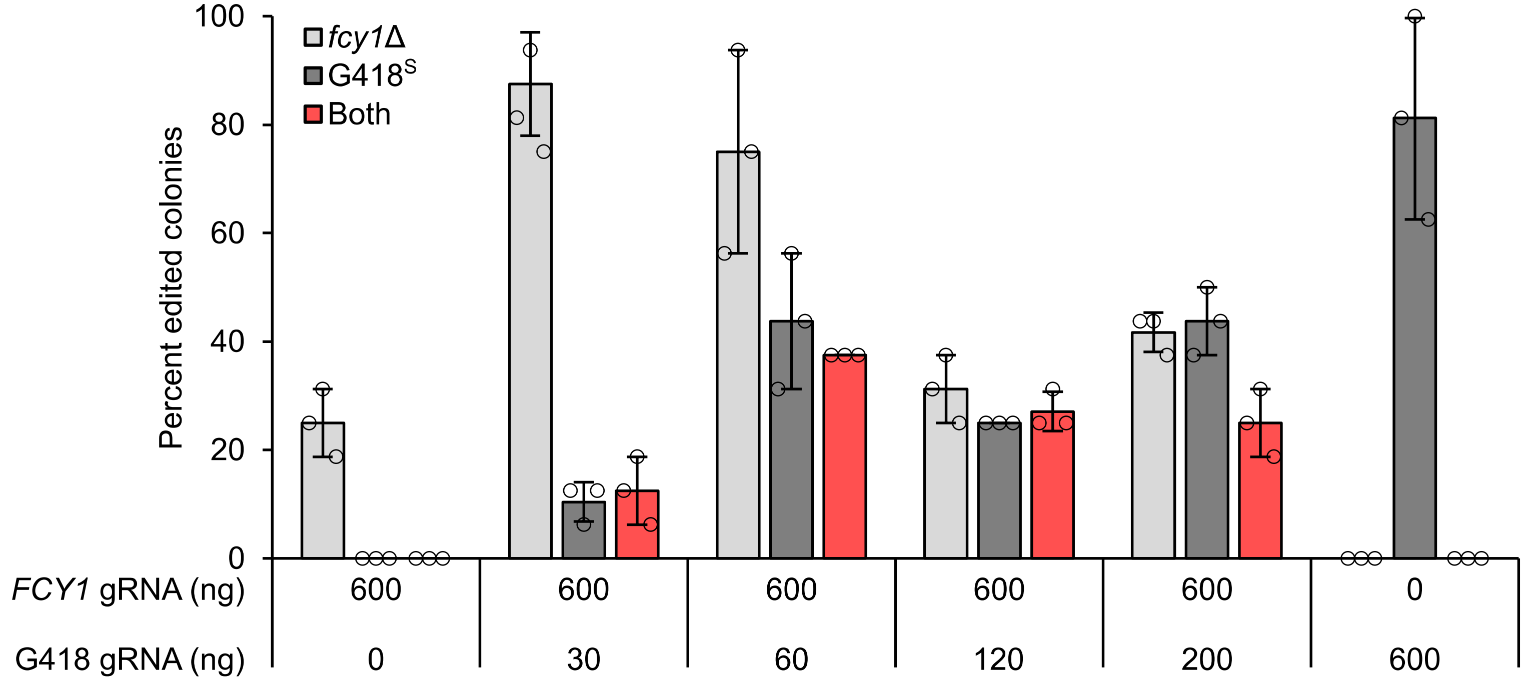

Supplementary Figure 8. Titration of *FCY1* and G418^{R} gRNA species. Varying amounts of G418 gRNA and 600 ng of *FCY1* gRNA were introduced to a G418^{R} *ade2*Δ mutant using pCas-Hyg-CEN6ARS4. Sixteen Hyg^{R} transformants from each condition were scored for *fcy1*Δ and G418^{S} by restreaking onto YPD plates containing 5-FC and G418, respectively. Error bars represent the mean ± s.d. of *n* = 3 independent biological samples. Source data are provided as a Source Data file.

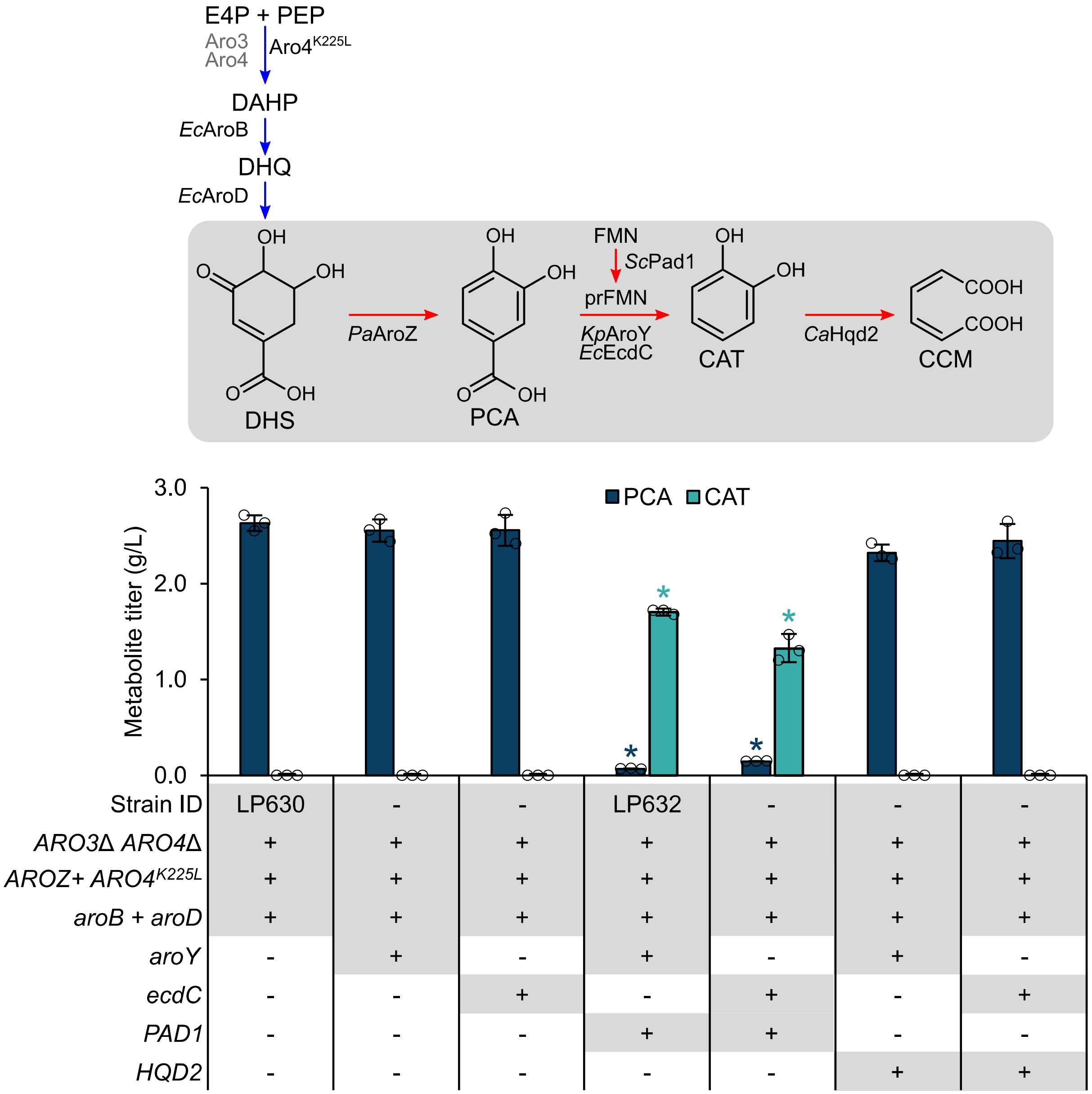

Supplementary Figure 9. Pad1 is required for PCA decarboxylase activity in engineered *P. occidentalis*. Combinations of PCA decarboxylase (*Kp*AroY or *Ec*EcdC), FMN prenyltransferase (*Sc*Pad1) and catechol dioxygenase (*Ca*Hqd2) were introduced to PCA-producing *P. occidentalis* (LP630). The native shikimate and heterologous CCM synthesis pathway are depicted in blue and red, respectively. Asterisks (*) denote a significant decrease in PCA titer or increase in catechol titer relative to the parent strain ($P < 0.05$). Statistical differences between parent and derivative strains were tested using two-tailed Student's *t*-test. Error bars represent the mean ± s.d. of $n = 3$ independent biological samples. Abbreviations: CAT, catechol; CCM, *cis*,*cis*-muconic acid; DAHP, 3-deoxy-D-arabinoheptulosonate 7-phosphate; DHQ, 3-dehydroquinate; DHS, 3-dehydroshikimate; E4P, erythrose 4-phosphate; FMN, flavin mononucleotide; PCA, protocatechuic acid; PEP, phosphoenolpyruvate; prFMN; prenylated flavin mononucleotide. Source data are provided as a Source Data file.

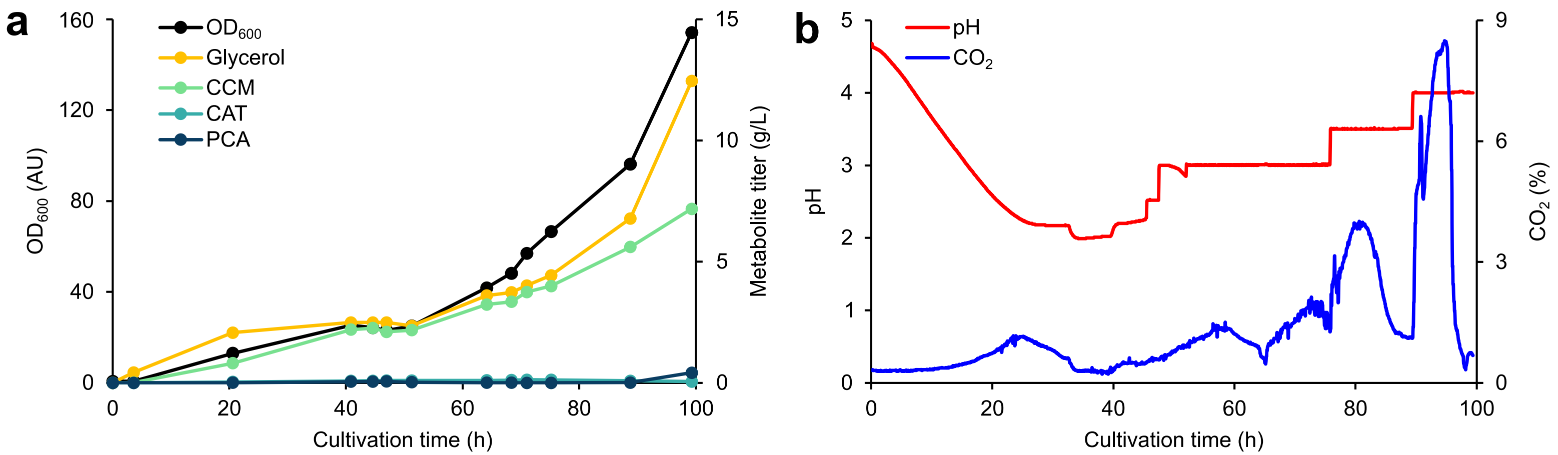

Supplementary Figure 10. Cultivation of a CCM-producing *P. occidentalis* strain (LP635) in a glucose-limited fed-batch fermentor at low pH. A CCM-producing strain (LP635) was fed 0.5 L of medium containing 360 g L^{-1} glucose. Biomass (OD_{600}) and heterologous product accumulation (**a**), and pH and CO_2 traces (**b**) are depicted. pH was adjusted manually using 4 N NaOH based on a decline in CO_2 production. Source data are provided as a Source Data file.

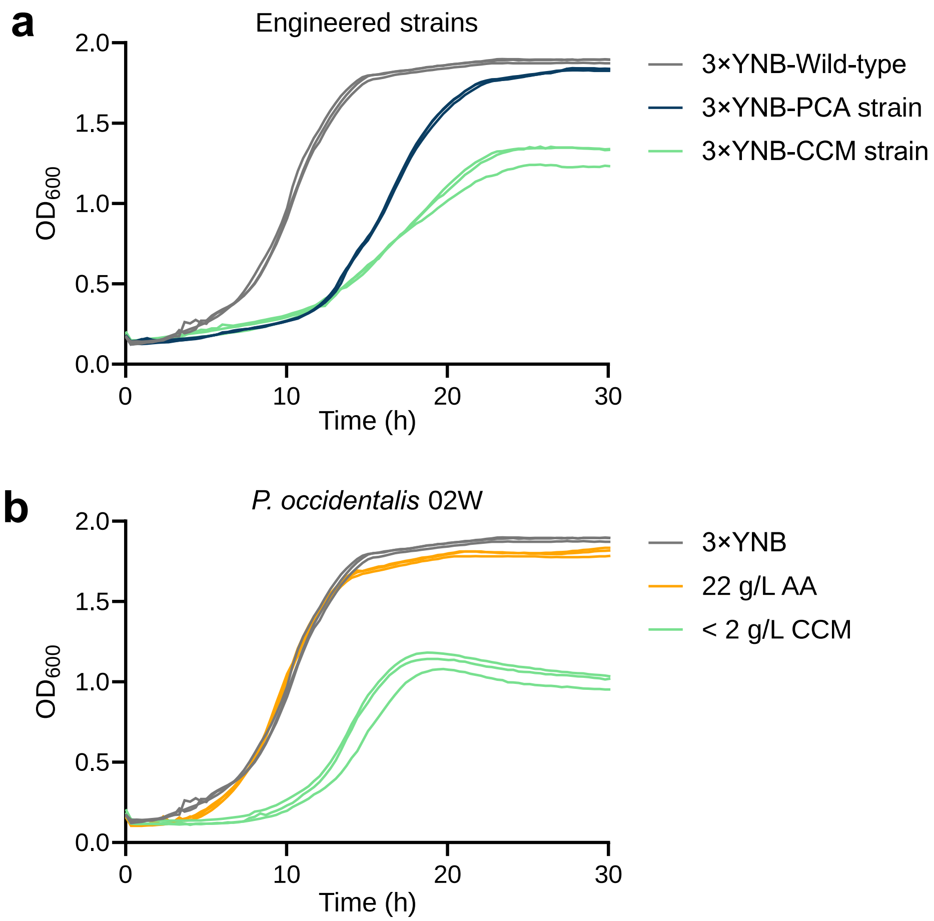

Supplementary Figure 11. Growth curves of engineered CCM-producing *P. occidentalis* and the wild-type strain supplemented with exogenous CCM. a, Growth of CCM-producing *P. occidentalis* and its PCA-producing precursor. Strains were cultivated in 3× YNB. **b,** Growth of wild-type *P. occidentalis* in 3× YNB saturated with adipic acid (22 g L^{-1} or 0.15 M) or CCM (< 2 g L^{-1}). n = 3 independent biological samples are overlaid for each strain and growth condition. Source data are provided as a Source Data file.

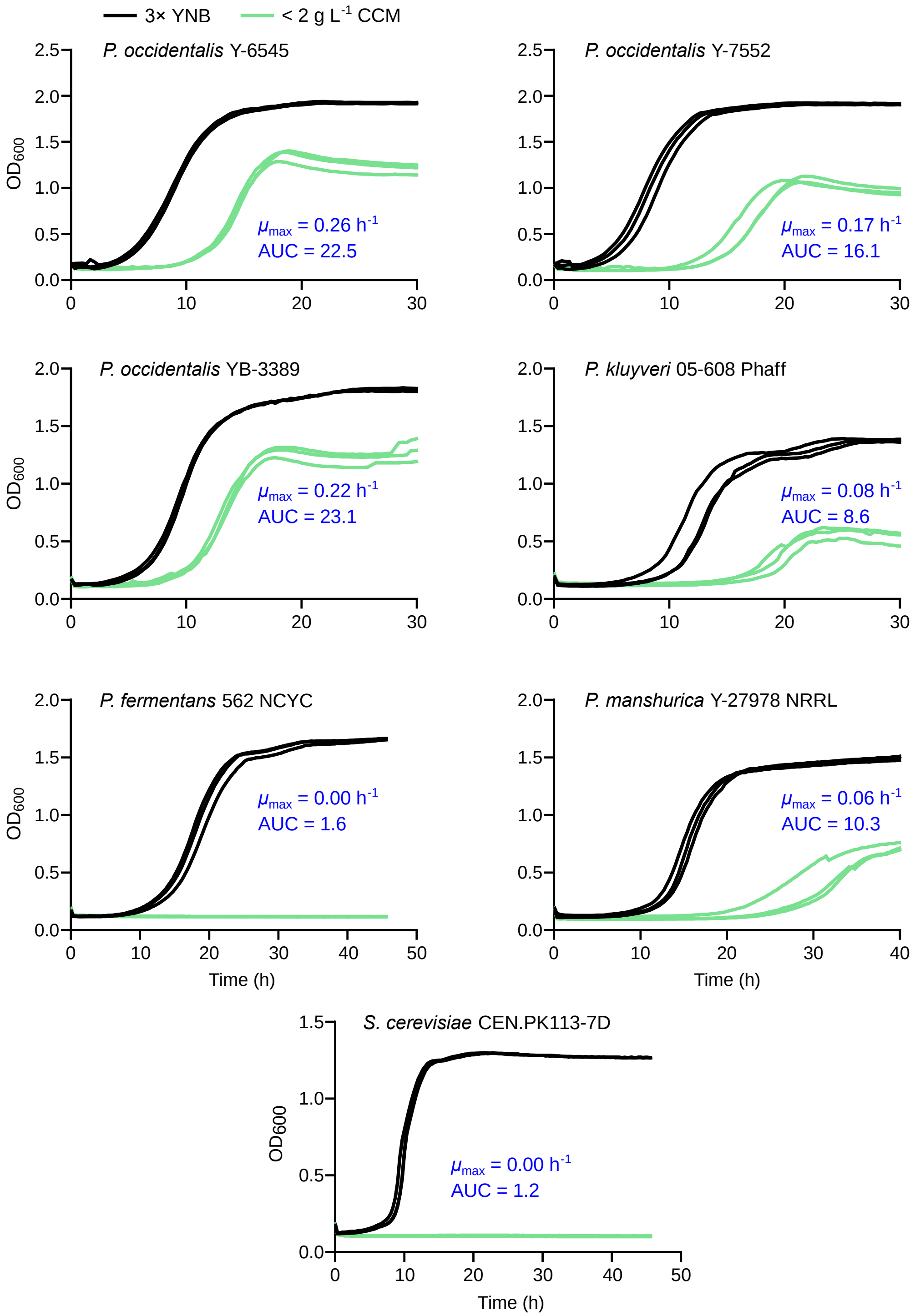

Supplementary Figure 12. Growth curves of adipic-acid-tolerant *Pichia* strains supplemented with exogenous CCM. 3× YNB was saturated with CCM (< 2 g L^{-1}). n = 3 independent biological samples are overlaid for each strain and growth condition. Maximum growth rate (μ_{max}) and area under the curve (AUC) are shown in blue for each strain grown in CCM. Source data are provided as a Source Data file.

Supplementary Table 1. Phenotypic overview of adipic acid tolerant *Pichia* species.[a]

**Characteristic**[b]	***P. occidentalis***	***P. kluyveri***	***P. manshurica***	***P. kudriavzevii***	***P. membranifaciens***	***P. fermentans***
Glucose fermentation	+	+	v	+	v	+
Galactose fermentation	-	-	-	-	-	-
Sucrose fermentation	-	-	-	-	-	-
Lactose fermentation	-	-	-	-	-	-
Glucose	+	+	+	+	+	+
Sucrose	-	-	-	-	-	-
Galactose	-	-	-	-	-	-
Lactose	-	-	-	-	-	-
Soluble starch	-	-	-	-	-	-
Cellobiose	-	-	-	-	-	-
L-Sorbose	v	-	v	-	v	-
L-Rhamnose	-	-	-	-	-	-
D-Xylose	-	v	-	-	v	+
L-Arabinose	-	-	-	-	-	-
D-Arabinose	-	-	-	-	-	-
Ethanol	+	+	+	+	+	+
Glycerol	+	+	v	+	v	+
Vitamin-free	+	-	v	+	v	-

[a] data obtained from Kurtzman *et al.* [1]

[b] growth data is provided under aerobic conditions unless stated otherwise

-, negative; +, positive; v, variable

Supplementary reference

1 Kurtzman, C., Fell, J. W. & Boekhout, T. The yeasts: a taxonomic study. (Elsevier, 2011).
