# Supplementary material for: Screening non-conventional yeasts for acid tolerance and engineering Pichia occidentalis for production of muconic acid
Source: Nat Commun. 2023 Aug 31;14:5294. doi: 10.1038/s41467-023-41064-5 (PMC10471774; doi:10.1038/s41467-023-41064-5)
Supplement: Supplementary file 3 — Description of Additional Supplementary Files [file 41467_2023_41064_MOESM3_ESM.pdf]

Description of Additional Supplementary Files

File name: Supplementary Data 1
Description: All 153 yeast strains ordered from public culture repositories.

File name: Supplementary Data 2
Description: Oligonucleotides utilized in this study.

File name: Supplementary Data 3
Description: Sequences of *Pichia occidentalis* promoters and terminators utilized in this study.

File name: Supplementary Data 4
Description: Sequences of *Pichia occidentalis* integration loci utilized in this study.

File name: Supplementary Data 5
Description: Genes and synthetic DNAs synthesized in this study.
